# Can a deprivation index be used legitimately over both urban and rural areas?

**DOI:** 10.1186/1476-072X-13-22

**Published:** 2014-06-14

**Authors:** Mélanie Bertin, Cécile Chevrier, Fabienne Pelé, Tania Serrano-Chavez, Sylvaine Cordier, Jean-François Viel

**Affiliations:** 1 INSERM-IRSET n° 1085, “Epidemiological Research on Environment, Reproduction and Development”, University of Rennes 1, Rennes, France; 2 Department of Epidemiology and Public Health, University Hospital, 2, rue Henri Le Guilloux, 35033 Rennes cedex 9, France

**Keywords:** Deprivation, Rurality, Urbanisation, Standardisation, Health need

## Abstract

**Background:**

Although widely used, area-based deprivation indices remain sensitive to urban–rural differences as such indices are usually standardised around typical urban values. There is, therefore, a need to determine to what extent available deprivation indices can be used legitimately over both urban and rural areas.

**Methods:**

This study was carried out in Brittany, France, a relatively affluent region that contains deep rural areas. Among the 1,736 residential census block groups (IRIS) composing the Brittany region, 1,005 (57.9%) are rural. Four deprivation indices were calculated: two scores (Carstairs and Townsend) developed in the UK and two more recent French measures (Havard and Rey). Two standardisation levels were considered: all of the IRIS and only the urban IRIS of the region. Internal validity (Kappa coefficients and entropy values) and external validity (relationship with colorectal cancer screening [CCS] attendance) were investigated.

**Results:**

Regardless of the deprivation measure used, wealthy areas are mostly clustered in the West and at the outskirts of major towns. Carstairs and Rey scores stand out by all evaluation criteria, capturing both urban and rural deprivation. High levels of agreements were found across standardisation levels (κ = 0.96). The distributions of deprivation scores were balanced across urban and rural areas, and high Shannon entropy values were observed in the capital city (≥0.93). Similar and significant negative trends were observed between CCS attendance and both deprivation indices, independent of the degree of urbanisation.

**Conclusions:**

These results provide support, despite potential sociological objections, for the use of a compromise index that would facilitate comparisons and interpretations across urban and rural locations in public health research.

## Background

Deprivation is measured over geographical areas rather than based on individual circumstances, assuming that inequalities are related not only to individual predisposing or behavioural factors but also to social and environmental influences at work above the level of the individual [1]. Deprivation indices capture both contextual properties of an area that are extrinsic to individuals (such as public amenities) and aggregated compositional properties of the individuals in the area. Contextual characteristics truly conceptualise areal phenomena, in opposition with compositional properties, which represent more of a sum of the characteristics of individuals in a given area [1,2], although interactions do occur between people and the social and physical resources in their environment [3]. First designed as an ecological solution to the absence of income/wealth indicators at an individual level [4,5], deprivation measures have since been used to explain health outcome inequalities [6-9], and, in addition to drive the health resource allocation process [10].

The composite scores of deprivation in general use are calculated using small-area census variables whose values are normalised or transformed, standardised to local, regional or national means, and combined according to a weighting scheme. These conventional area-based deprivation indices are designed to measure material, rather than social deprivation (e.g., isolation) [11]. More recent indicators have been developed using principal component analysis (PCA) [12,13].

One of the main limitations of deprivation indices is that they are usually sensitive to urban–rural differences. The original focus on material deprivation has generated deprivation indicators standardised around typical urban values that may not be appropriate for rural areas [11,14,15]. These indices typically generate the same geographical pattern, with the worst scores in larger urban centres and the most favourable scores in rural areas, particularly in affluent rural commuter settlements close to large urban centres [11]. Some conceptual and methodological constraints therefore need to be considered when estimating deprivation for both urban and rural areas, namely indicator component relevance, population heterogeneity, and the level of standardisation.

Indicators that capture deprivation and hardship in cities may not perform as well in the countryside, as some issues (e.g., resource, opportunity and mobility deprivations) can disproportionally affect rural people [16]. Factors such as physical and social isolation and declining levels of the provision of services (including health services) can together make up a major component of deprivation that is unfortunately often overlooked by conventional indices. Some material indicators (car ownership, housing tenure), acting as proxies for income in urban population, are weaker discriminators of wealth in rural settings [14]. People in rural areas with low incomes make sacrifices to keep a car because of longer travel distances and poor public transportation [17], and housing tenure has been shown to be a biased measure of deprivation in the rural context [18]. Conversely, the unemployment rate can be considered a good marker of social deprivation in rural areas, especially at the scale of small geographical units [7,18,19].

Rural populations are more heterogeneous than their urban counterparts, with some of the poorest people interspersed amongst very wealthy landowners, commuters, and professionals [8]. These pockets of disadvantage in the countryside, hidden by favourable but meaningless averages, may confer health disadvantages that may not be gauged by urban-biased measures. Conversely, rural areas are perceived to have more supportive social networks than urban areas and may in turn be more advantaged in terms of health outcomes [20].

The issue of the standardisation process has been addressed only to a limited extent thus far, despite its obvious importance. Urban bias is inherent in the national standardisation of deprivation indices, which overlooks aspects of social disadvantage unique to rural environments [11]. Moreover, conventional indices reflect relative deprivation as individual, small-area scores are affected by values in the remaining parts of the county, region, or country under scrutiny and depend on the geographical extent of the study area (with potentially variable degrees of urbanisation among study areas).

Rather than considering separate ways of measuring deprivation in urban and rural areas, it seems more appropriate to use a single index across a given region, to facilitate comparison, updating and interpretation. It would therefore be useful to determine to what extent available deprivation indices can be used legitimately over both urban and rural areas. To achieve this goal, we examine the sensitivity of several indices to the standardisation process, and we determine whether these indices demonstrate associations with health need across the urban–rural spectrum.

## Methods

### Study area

The Brittany region (27,209 km^2^, 3,175,064 inhabitants in 2009) occupies a large peninsula in northwest France, with rural areas accounting for 55.93% of the surface area.

The smallest geographical census units available in France were considered. These so-called “IRIS”, defined by the National Institute for Statistics and Economic Studies (INSEE), are equivalent to US census block groups or British lower super output areas. The regional capital and towns with more than 5,000 inhabitants are divided into several IRIS, while smaller towns form one IRIS each for a total of 1,797 IRIS in Brittany. Sixty-one IRIS classified as industrial zones, leisure parks or commercial zones were further excluded. Consequently, the study area comprises a total of 1,736 residential IRIS, each with relatively homogeneous social characteristics.

### Deprivation indices

Four deprivation indices were considered and calculated from 1999 census data: two scores (Carstairs [4] and Townsend [5]) developed in the UK and commonly used in international epidemiological studies, and two more recent French deprivation indices [12,13]. Briefly, the Carstairs and Townsend indices are composite, additive combinations of census-derived variables, while the other two indices were generated using PCA applied to sets of from 4 to 19 original variables (Table 1).

**Table 1:** Census-derived variables contributing to the four deprivation indices under study

**Variables**	**Townsend**	**Carstairs**	**Havard**	**Rey**
Unemployment rate	X	X	X	X
Proportion of unemployed people > 1 y			X	
Proportion of households without a car	X	X	X	
Proportions of households with ≥ 2 cars			X	
Primary residences with > 1 person/room	X	X	X	
Mean number of people/room			X	
Blue-collar workers in the labour force		X	X	X
People ≥ 15 years old with only elementary education			X	
People ≥ 15 years old with at least high-school diploma			X	X
People ≥ 15 years old with university graduation			X	
People with permanent work contracts			X	
People with non-permanent work contracts			X	
Households owners of their primary residence	X		X	
Subsidised housing among primary residences			X	
Primary residences that are houses or farms			X	
Primary residences that are multiple-dwelling units			X	
Single-parent families			X	
Foreigners in total population			X	
Median income per consumption unit or household			X	X

### Degree of urbanisation

**Figure 1:**
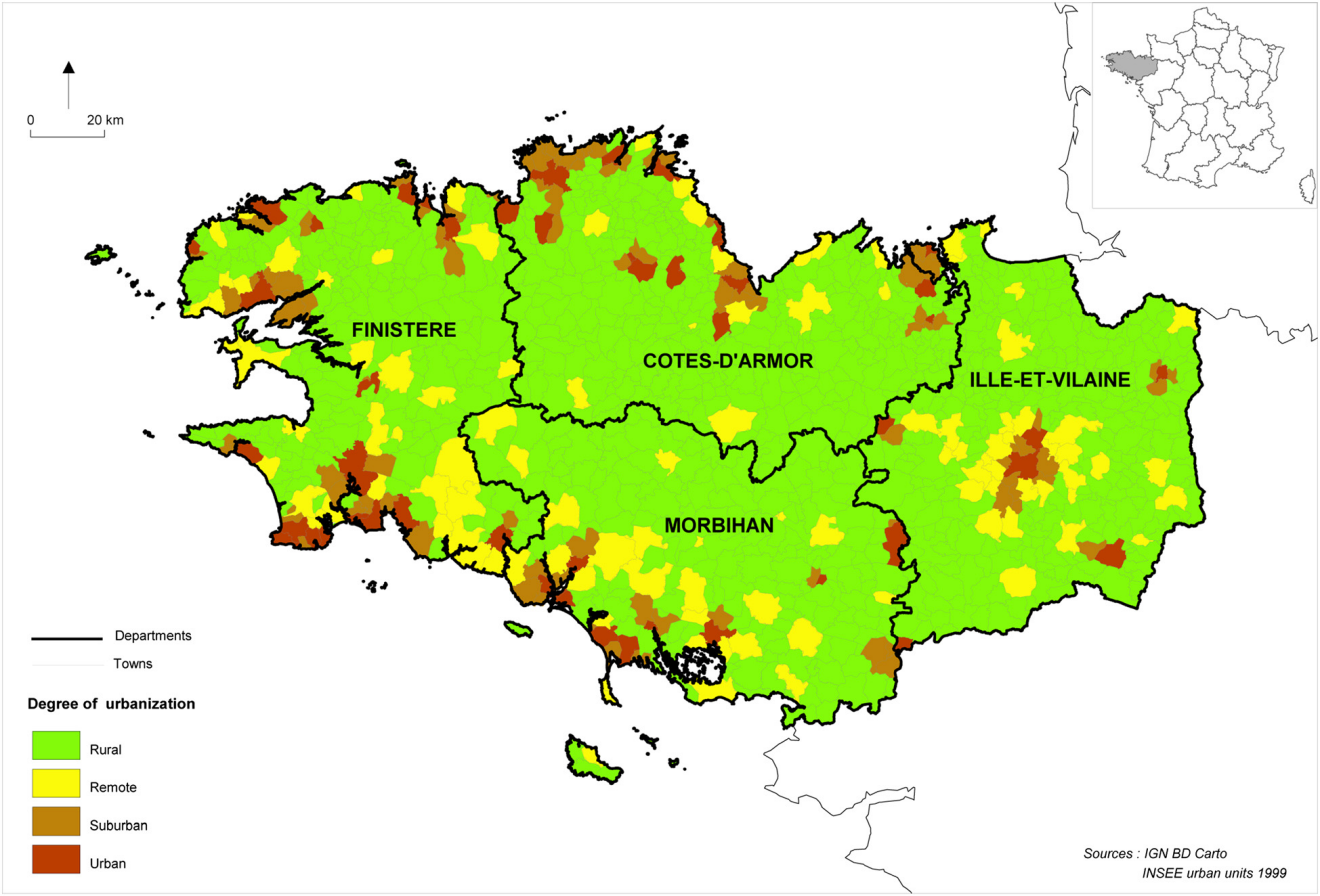
Degree of urbanisation of Brittany towns (Brittany, France, National Institute for Statistics and Economic Studies).

According to INSEE, an “urban unit” is a town or a group of towns that includes a built-up area of at least 2,000 inhabitants and in which no building is farther than 200 m away from its nearest neighbour. Urban units are further categorised as “highly urban”, “suburban”, or “isolated” towns depending on the number of towns involved (and their respective populations). All other towns are considered “rural”. Each IRIS was assigned the degree of urbanisation of the town it belonged to according to this classification scheme (Figure 1). For most analyses, highly urban, suburban and isolated IRIS were aggregated into a single “urban” category.

### Standardisation levels

First, deprivation indices were calculated on all of the IRIS (corresponding to a process hereafter called “overall standardisation”). Second, for sensitivity analyses, the only urban IRIS of the study region served as reference in the standardisation process (hereafter called “urban standardisation”). Consequently, for each rural IRIS, each variable contributing to the Townsend or Carstairs scores was standardised by subtracting the mean of the urban IRIS from the value of that variable for the rural IRIS considered, then dividing by the standard deviation estimated from the urban IRIS. For PCA-based indices, PCA was carried out on the only urban IRIS (active observations), while rural IRIS were introduced as supplementary observations and projected onto the principal components calculated using data for the urban IRIS.

### Internal validity

Quadratic weighted kappa statistics (κ) (using the quintiles of deprivation scores) were calculated for quantifying agreement between the two standardisation level estimates for each deprivation index [21].

Shannon entropy was calculated to assess the ability of each deprivation index to discriminate among quintiles of deprivation in a specific area [22]. The logarithm base 5 was used to generate a Shannon index that varied between 0 and 1. However, the entropy could not be assessed on the whole region (on which the quintiles were estimated) because, by definition, quintiles are five equally numerous subsets and the Shannon index hence would take the maximum value. We chose to estimate Shannon entropy on the capital city of Rennes (84 IRIS, Figure 1), the more densely urbanised area of the study region. Low entropy values would reflect an unequal deprivation distribution between IRIS, highlighting an urban bias (the vast majority of the IRIS falling in the upper quintile of the deprivation score considered).

### External validity

Associations between inequalities in healthcare utilisation and socio-economic deprivation are well established [19]. Previous studies have shown a relationship between socio-economic factors and neighbourhood deprivation, and colorectal cancer screening (CSS), with the most deprived areas exhibiting the lowest attendance rates [23,24].Age and sex standardised CCS attendance rates (age 50 to 75 years, 2009–2010) were made available at the IRIS level from a population-based screening programme implemented in Ille-et-Vilaine (I&V) (514 IRIS), one of the four departments of which Brittany is composed (CSS data were unfortunately not available at the IRIS level in the remaining departments) (Figure 1).

Spatial regressions models were initially performed with CCS attendance rate as the dependent variable and deprivation quintile as the categorical, independent variable. The regression diagnostics revealed no spatial autocorrelation and guided us towards non-spatial linear regression models [25]. Simple and multiple (by adjusting for the degree of urbanisation) linear regressions were then performed introducing in turn the four deprivation indices that were based on the overall standardisation method. P-trend values were calculated across quintiles. All statistical tests were two-tailed, and P values below 0.05 were considered statistically significant.

All analyses were performed with STATA SE version 12 (USA, College Station, TX: StataCorp LP), R version 2.15.0 (Vienna, Austria: R Development Core Team) with FactoMineR package [26] and GeoDa version 1.3.27 (USA, Urbana-Champaign, IL: University of Illinois).

## Results

### Characteristics of the IRIS

Among the 1,736 residential IRIS composing the Brittany region, 341 (19.6%) are highly urban, 176 (10.1%) are suburban, 214 (12.4%) are isolated, and 1,005 (57.9%) are rural. Their median population and median surface area are 1654.5 inhabitants and 11.7 km^2^, respectively.

### Deprivation indices

Descriptive statistics of the four deprivation indices divided into quintiles (defined using the full distribution for each index) are reported across the level of standardisation and the degree of urbanisation (Table 2). Imbalance between urban and rural deprivation is observed for both Townsend and Havard indices calculated using the overall standardisation method. More urban IRIS are classified in the most deprived quintile (34.75% and 42.09%, respectively) than are their rural counterparts (9.25% and 3.74%, respectively). Similar patterns are observed for the urban standardisation but with somewhat narrower differences. In contrast, using the Rey score, more urban IRIS are classified in the wealthiest quintile (34.66% and 32.83% with overall and urban standardisation, respectively) than their rural counterparts (9.2% and 10.75% with overall and urban standardisation, respectively). As a result, the Carstairs index appears to be the most balanced, and, therefore, the less sensitive to the urbanisation degree.

**Table 2:** **Distribution of deprivation quintiles**^**a **^**by deprivation indices (%) according to the level of standardisation and the degree of urbanisation (731 urban IRIS and 1005 rural IRIS, Brittany, France)**

	**Overall standardisation**		**Urban standardisation**	
	**Urban areas**	**Rural areas**	**Urban areas**	**Rural areas**
Townsend				
Q1^b^	17.8	21.7	18.7	21.0
Q2	15.2	23.5	16.7	22.4
Q3	15.9	23.0	16.7	22.4
Q4	16.4	22.6	16.6	22.5
Q5	34.8	9.3	31.3	11.7
Carstairs				
Q1	27.9	14.3	29.4	13.2
Q2	18.7	20.9	20.8	19.4
Q3	17.9	21.5	18.3	21.2
Q4	17.0	22.2	16.4	22.6
Q5	18.5	21.1	15.1	23.6
Havard				
Q1	13.2	25.1	23.5	17.5
Q2	11.7	26.1	15.2	23.5
Q3	11.1	26.5	11.9	25.9
Q4	21.9	18.6	13.5	24.7
Q5	42.1	3.7	35.8	8.5
Rey				
Q1	34.7	9.2	32.8	10.8
Q2	24.4	16.8	24.2	16.9
Q3	19.7	20.2	17.4	21.9
Q4	10.9	26.7	12.9	25.2
Q5	10.5	27.1	12.7	25.3

^a^Calculated from all of the 1736 IRIS.

^b^Q1 represents the wealthiest quintile and Q5 the most deprived quintile.

### Internal validity

**Figure 2:**
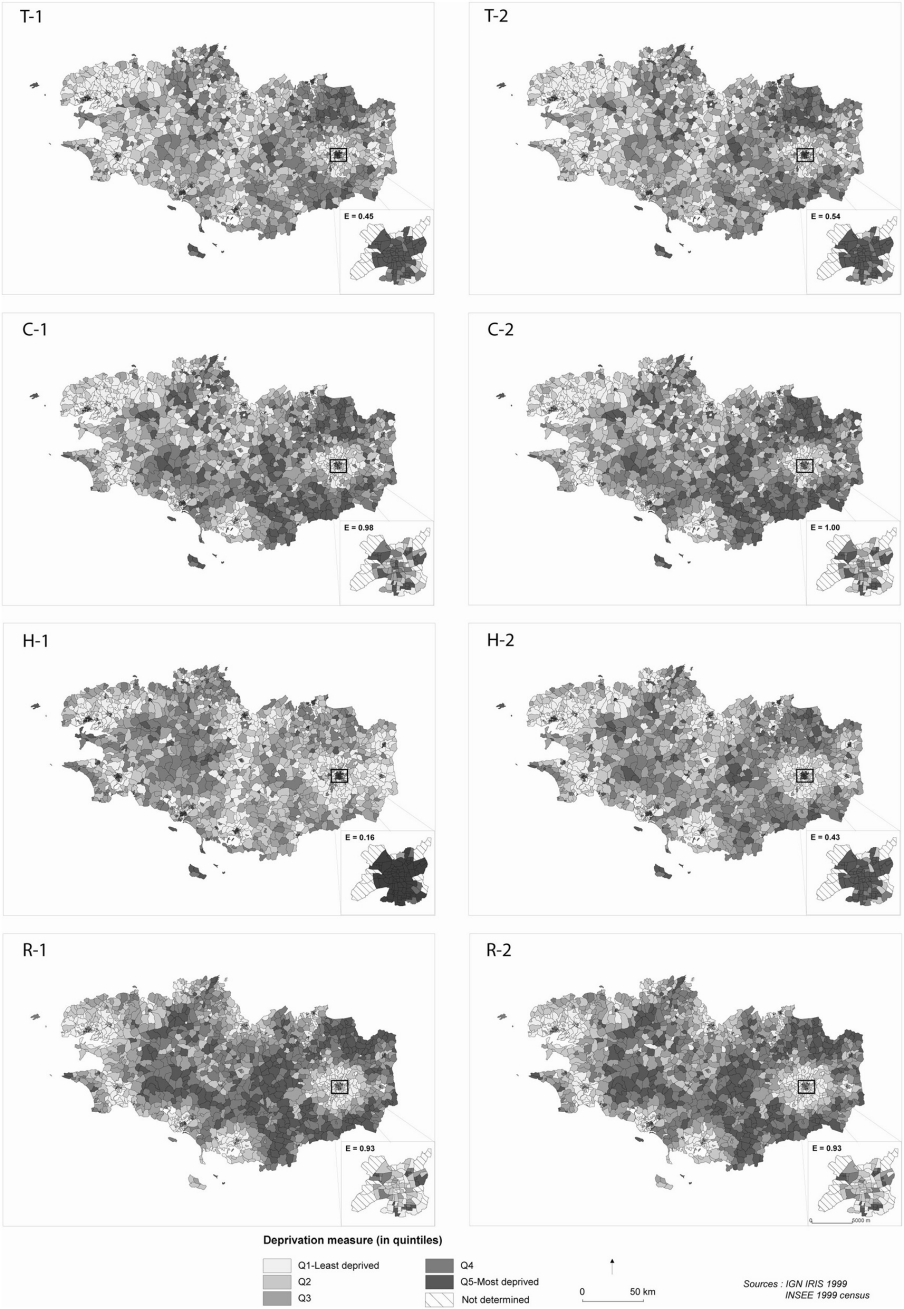
**Deprivation indices by IRIS (1999, Brittany, France).** Inset maps focus on the capital city of Rennes (84 IRIS) and their measures of entropy (E). T: Townsend, C: Carstairs, H: Havard, R: Rey. 1: “Overall standardisation”, 2: “Urban standardisation”.

The distribution of deprivation across geographic space, by standardisation level and deprivation index, is presented in Figure 2. Inset maps focus on the capital city of Rennes, for which entropy values were estimated. These maps show considerable diversity among IRIS. Regardless of the measure of deprivation used, wealthy areas are mostly clustered in the West and at the outskirts of the major towns. The most deprived IRIS are located in the North East and South East. Visual inspection of these maps across standardisation levels shows a larger heterogeneity for Havard score, which is confirmed by a lower value of κ (0.78) compared to the other three scores, which exhibit very high levels of agreement across standardisation levels (κ = 0.96).

When focusing on the most densely populated area of the region (Rennes city), one striking cartographic feature is the discriminating capacity of both the Carstairs and Rey indices. Conversely, inset maps are overwhelmed by (black) areas found to be deprived using Townsend and Havard measures. Entropy values objectively characterise this cartographic approach. Very high entropies (≥0.93) are observed for both Carstairs and Rey scores across standardisation levels, whereas entropy values appear much lower (≤0.54) and broader across standardisation levels for the remaining two indices.

### External validity

**Figure 3:**
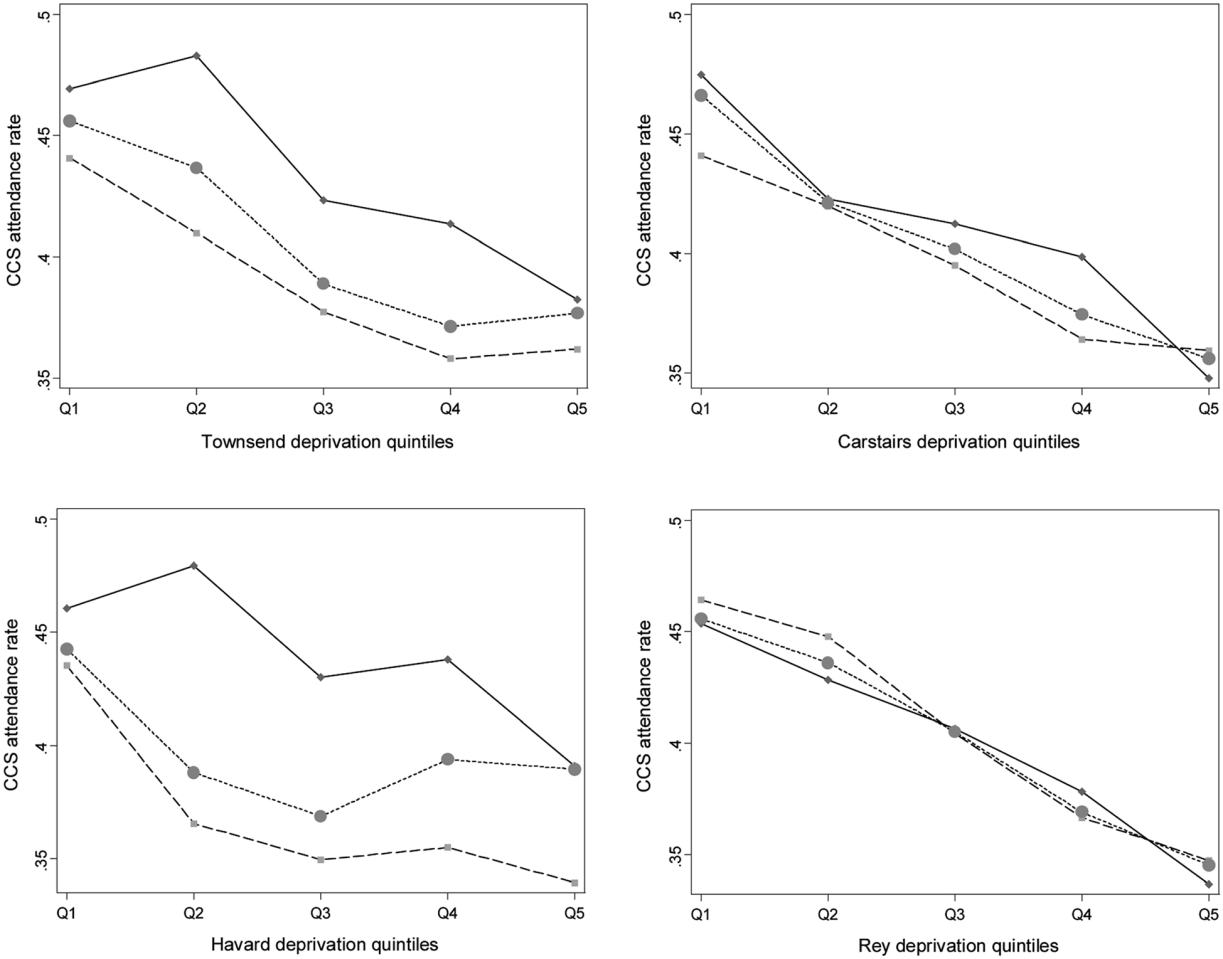
Colorectal cancer screening (CSS) attendance rates according to deprivation scores (quintiles), and the degree of urbanisation (urban, rural, all IRIS) (solid lines: urban areas; dashed lines: rural areas; dotted lines: all areas).

Large variations in the CCS attendance rate were found in I&V department, ranging from 14.2% to 71.5%, for an average of 39.9%. Figure 3 displays CCS attendance rates according to deprivation scores (quintiles) and the degree of urbanisation (urban, rural, and global). On the whole, attendance rates decrease linearly with deprivation scores. Almost superimposable linear regression lines are highlighted for every urbanisation status, when using the Carstairs or Rey indices. Greater variability between regression lines is observed when using Townsend and Havard indices, higher CCS attendance rates being observed in urban IRIS at all deprivation levels. Regression modelling confirms these graphic features (Table 3). All regression coefficients are negative and statistically significant. The best goodness-of-fits are found for the Rey and Carstairs indices (R^2^ = 0.216, and R^2^ = 0.170, respectively); both coefficients of determination remained unchanged after adjustment for the degree of urbanisation (R^2^ = 0.217 and R^2^ = 0.176, respectively).

**Table 3:** Colorectal cancer screening attendance rate regressed against deprivation indices (514 IRIS, Ille-et-Vilaine, Brittany, France)

	**Slope**	**P-trend**^**a**^	**R**^**2b**^
*Univariate analysis*			
Townsend	−0.021	< 10^−14^	0.100
Carstairs	−0.027	< 10^−21^	0.170
Havard	−0.011	< 10^−14^	0.033
Rey	−0.029	< 10^−26^	0.216
*Multivariate analysis*^*c*^			
Townsend	−0.023	< 10^−15^	0.157
Carstairs	−0.025	< 10^−17^	0.176
Havard	−0.023	< 10^−17^	0.144
Rey	−0.030	< 10^−21^	0.217

^a^P-trend across deprivation quintiles.

^b^R^2^: coefficient of determination.

^c^Adjusted for the degree of urbanisation (urban vs. rural).

## Discussion

This study provides evidence that among four indices considered, two (Carstairs and Rey) appear sensitive to disadvantage across the range of location types, independent of the standardisation process.

The strengths of this study arise from the study area, the geographical scale of analysis, the urbanisation description, the sensitivity analysis with respect to the standardisation level, and the external validity assessment. First, the Brittany region is on average relatively affluent but yet has deep rural areas, making it an ideal study area of sufficient population size to check whether deprivation indices can be used fairly over both urban and rural areas.

Second, deprivation was measured at the most detailed scale of analysis possible using French census data (the median population of an IRIS is 1654.5 inhabitants), leaving few pockets of deprivation masked by area-based averages and allowing some key aspect of rural deprivation to be gauged using some standard indicators.

Third, there are many ways to quantify urbanisation (population density, proximity to urban settings, economic activities, sociocultural characteristics, etc.). As in many European countries, the definition of French urban area is based on population cores represented by a continuous, built-up zone around an urban centre. The resulting classification scheme has the advantages of recognising the continuum and integration between urban and rural and of taking into account both population sizes and geographical patterns. Although much of the analysis was undertaken using an urban–rural dichotomy, our results show that urban–rural diversity was still captured at the local level.

Fourth, we carried out a sensitivity analysis for this region-based study. The natural inclination would have been to use supra-level (i.e., national) standardisation (and its inherent urban bias), as standardisation levels are usually assimilated to larger geographical extents [11]. We instead chose to rely on infra-level standardisation to avoid further heterogeneity factors (differences in economic development, diet, lifestyle and other socio-cultural factors) while still standardising around typical urban values.

Fifth, extrinsic relevance of deprivation scores was assessed using a measure of poorer access to health services as an indicator of need, demonstrating how the association of various deprivation scores with CCS attendance might vary across diverse urban–rural geographies.

Although care was taken to reduce the potential for bias, this study was subject to some limitations. This ecological study (although based on small areas) shares the limitations of all such studies. All deprivation scores were area-based and we were unable to distinguish between the influence of the surrounding areas (contextual effects) and the influence of the individuals within an area (compositional effects), both of which may contribute to spatial inequalities in socio-economic welfare and health [1,2]. A multi-level analysis, including both individual and area estimates of deprivation, could not be carried out as individual census data were not available due to strict legal constraints.

Finally, our - admittedly limited - attempts to explore the identification and measurement of rural deprivation only encompassed four deprivation measures although many more metrics have been described worldwide.

Analysing rurality and deprivation remains a challenge because of complex interplay between factors associated with income, social circumstances, access to services, and choice. Addressing some of these issues, this study meets the needs of identifying valid and measurable indicators of both urban and rural deprivation and testing their utility in determining any relationship with health care need across the urban–rural spectrum [27].

Two deprivation measures stand out by all evaluation criteria, capturing similar deprivation trends across a large geographical area containing a diverse population: the Carstairs and Rey scores. The standardisation level had no influence (high κ values) on deprivation trends, distributions of deprivation scores across urban and rural areas were balanced, and high entropies were observed in the capital city (revealing the ability of these scores to distinguish different deprivation levels at a sub-city scale). Similar negative linear trends were observed between CCS attendance and both deprivation scores, providing a consistent representation of healthcare utilisation across the urban–rural continuum. Moreover, adjustment for the degree of urbanisation left goodness-of-fit statistics unchanged. Both results support the hypothesis that the underlying relation between health and deprivation is the same in rural areas as in urban areas, provided rural deprivation is properly assessed [7,8,28].

A composite deprivation measure is based on a theoretical background, a number of census variables, and an ad-hoc weighting scheme. Therefore, any attempt to explain its overall performance by considering its components individually should be cautious as exemplified by the key observation of Gilthorpe and Wilson [19]. They clearly showed that constituent components may behave differently and yield considerable perturbation in relation to health care utilisation across the urban–rural spectrum while the composite deprivation measure may not. In a tentative explanation for the consistencies of Carstairs and Rey indices, we simply note that low social class, one common component of these indices (and absent from the Townsend score) has been shown to be representative of deprivation across the urban–rural spectrum [7,18,19].

## Conclusions

Our results provide support, despite potential sociological objections, for choosing a compromise deprivation index that would facilitate comparisons and interpretations across urban and rural locations in public health research. If a unique score is to be chosen, two practical considerations favour the Carstairs index: its widespread use in social and environmental epidemiology allowing international comparisons, and the simplicity of its calculation (unweighted sum in contrast to PCA for the Rey score). However, more work is needed to test the relevance of the Carstairs and Rey indices in other mixed urban–rural regions and against different measures of health need, and to consider other indicators of deprivation.

## Abbreviations

CCS: Colorectal cancer screening; PCA: Principal component analysis.

## Competing interests

The authors declare that they have no competing interests.

## Authors’ contributions

JFV, MB, CC and SC have contributed to the conception and design of this paper. Analyses were undertaken by MB and supervised by JFV. CC, FP, TSC, and SC have contributed to the interpretation of the findings, drafts and revisions of the paper. MB, CC, FP, TSC, SC and JFV have approved the final version of this manuscript.
